# Risk of Perihippocampal Recurrence After Hippocampal Avoidance in Prophylactic Cranial Irradiation: A Literature Review

**DOI:** 10.7759/cureus.86007

**Published:** 2025-06-14

**Authors:** Georgios Giakoumettis, Areti Gkantaifi, Dimitrios Giakoumettis, Konstantinos Kouskouras, Anastasios Siountas, Panagiotis Bamidis, Emmanouil Papanastasiou

**Affiliations:** 1 Laboratory of Medical Physics and Digital Innovation, School of Medicine, Faculty of Health Sciences, Aristotle University of Thessaloniki, Thessaloniki, GRC; 2 Laboratory of Medical Physics and Digital Innovation, American Hellenic Educational Progressive Association (AHEPA) University Hospital, Thessaloniki, GRC; 3 Department of Radiation Oncology, School of Medicine, Faculty of Health Sciences, Aristotle University of Thessaloniki, American Hellenic Educational Progressive Association (AHEPA) University Hospital, Thessaloniki, GRC; 4 Neurosurgery, Agios Savvas, General Anticancer Oncological Hospital of Athens, Athens, GRC; 5 Radiology Clinic, School of Medicine, Faculty of Health Sciences, Aristotle University of Thessaloniki, American Hellenic Educational Progressive Association (AHEPA) University Hospital, Thessaloniki, GRC

**Keywords:** failure, hippocampal avoidance, pci, prophylactic cranial irradiation, radiotherapy, small-cell lung cancer

## Abstract

A standard practice in the treatment of patients with small-cell lung cancer (SCLC) is prophylactic cranial irradiation (PCI) to reduce the chance of brain metastases. However, whole brain radiation therapy (WBRT) has been associated with concerns about neurocognitive decline. This has led to the development of WBRT techniques with the simultaneous avoidance of the hippocampus (HA). This article reviews the existing literature on the incidence of hippocampal failure after HA PCI in patients with SCLC. The effort to protect the hippocampus aims to reduce side effects at a cognitive level, but, as reported in various studies, the results regarding safety and effectiveness are ambiguous. Some indicate a higher risk of recurrence in the hippocampal and perihippocampal regions, particularly in non-oligometastatic patients. Despite any concerns, many trials have shown that HA in PCI significantly reduces cognitive decline without compromising overall survival or control of brain metastases. The mixed results noted between studies indicate the necessity of clinical trials to elucidate the benefits and risks of PCI with simultaneous hippocampal protection in patients suffering from SCLC.

## Introduction and background

Radiotherapy constitutes one of the most effective treatment options for solid malignancies. Data from cancer statistics indicate that 234,580 new cases of bronchus and lung cancer would occur in the United States in 2024. Small-cell lung cancer (SCLC) malignancies typically account for around 13-15% of all lung cancer cases, which suggests approximately 30,000 to 35,000 SCLC cases [1]. Limited disease concerns one-third of cases, and the treatment includes combined chemoradiotherapy with prophylactic cranial irradiation (PCI). The management of extensive disease, referring to two-thirds of cases, includes chemotherapy and thoracic radiotherapy primarily as a consolidation. In case of at least partial response, patients may also receive PCI. SCLC is a highly aggressive disease with a high tendency to spread to the brain [2]. About 10-14% of SCLC cases present brain metastases at the time of diagnosis, while half metastasise to the brain during disease progression [3,4].

Due to the high propensity of developing brain metastases, several researchers have investigated the potential benefit of PCI. One meta-analysis demonstrated improved control by decreasing the incidence of intracranial failure and overall survival benefit with the use of PCI in limited-stage (LS) SCLC patients [5], while limited data indicate that there is also a survival benefit by delivering PCI in patients with extensive-stage SCLC [6].

Despite the benefit of PCI, there is great concern about the potential radiation toxicity. Characteristically, regarding acute side effects, symptoms may include fatigue, nausea, headache, changes in appetite, and hair loss. In contrast, data concerning late toxicity illustrate that PCI may be related to neurocognitive impairment, including short-term memory, ataxia, weakness, and communication deficit, which has a negative impact on a patient’s quality of life [7-9]. The pathogenesis of neurocognitive dysfunction is potentially attributed to microangiopathy and, consequently, microvascular ischemia caused by radiation or impairment of critical brain centers, including the hippocampus [10]. The latter is highly sensitive to radiotherapy, and even a low dose of ≤2 Gy can significantly cause short-term memory decline [11]. Because a total PCI dose of 36 Gy was associated with an increased risk of developing chronic neurotoxicity, the standard dose for patients with LS SCLC who achieve complete response after chemoradiotherapy is 25 Gy in 10 daily fractions [7].

Additionally, the need to preserve neurocognitive function is highlighted by the fact that this is already impaired in SCLC patients and adversely affected by whole-brain radiotherapy (WBRT), specifically in older patients [12]. Consequently, these data indicate the need to take initiatives to reduce treatment-related effects. As the brain compartment for memory specificity is located on the hippocampal gyrus, much effort is made in the reduced dose to this region during WBRT to prevent neurocognitive decline [13].

As a result, a major scientific interest is observed in the therapeutic strategies capable of minimizing neurotoxicity in PCI. Neuroprotective agents could potentially reduce adverse effects on neurocognitive function and have an influential role in limiting the risk of cognitive decline. A benefit regarding later cognitive impairment after WBRT was illustrated in one prospective study, without achieving statistical significance at 24 weeks follow-up (p = 0.059) [14]. A promising strategy attracting great scientific interest is WBRT or PCI with hippocampal avoidance (HA), given the great contribution of the hippocampus to memory function. Thanks to the rapid technological development in radiation oncology and the wide availability of newer techniques in radiotherapy departments, HA PCI can be enabled. Indeed, the potential benefits of HA PCI and WBRT are under investigation in many institutions and are even used in daily routines in some. Recently, a randomized phase III trial reported improved neurocognitive function in favor of HA WBRT after six months in 518 patients (NRG Oncology CC001) [15].

However, ambiguities arise about the technique of HA PCI in SCLC patients, given the risk versus the potential benefit. Given that the literature data are conflicting about the incidence of intracranial failure after HA PCI/WBRT, there is a great need to enroll patients in randomized clinical trials to extrapolate valuable results about the benefit of this novel approach in SCLC patients. Therefore, we considered it fruitful to conduct this review summarizing the published literature about the risk of recurrence in the HA region after PCI in SCLC patients, aiming to extrapolate potential conclusions given the distribution of metastatic lesions in relation to the hippocampus potentially defining the zone of milimeters around the hippocampus which is at greater risk for recurrence.

## Review

Methodology

This study is a literature review. Given that existing published literature was included, no ethical approval was sought. Papers offering any data concerning the risk of developing hippocampal metastasis after PCI or WBRT with HA in patients with SCLC were included and evaluated in the present literature review. Two independent researchers conducted a systematic literature review using the PubMed database and Google Scholar search to gather journal articles. These sources are among the most commonly utilized in the medical literature following the Preferred Reporting Items for Systematic Reviews and Meta-Analyses (PRISMA) guidelines. [16]. The results were narrowed by selecting human articles published from January 1, 2002, to January 31, 2025. Only publications published in the English language were selected.

Furthermore, the abstracts from search results were screened to determine eligibility for inclusion in the review, and additional references were selected from relevant articles. The inclusion criteria were any type of article illustrating the incidence of metastasis in the HA area in SCLC patients. The search terms included “Small Cell Lung Cancer,” “Radiotherapy,” “Hippocampal Avoidance,” “Prophylactic Cranial Irradiation,” and “Failure.” The search yielded 118 citations, and 18 studies that fit the inclusion criteria were included. A filter was not used as any study type was considered without restrictions on randomized controlled trials. The study selection included observational, prospective, comparative, randomized, double-blind, placebo-controlled or uncontrolled, and retrospective studies and systematic reviews and meta-analyses. Cross-references from the included studies were hand-searched. Two independent reviewers retrieved, printed, and manually reviewed all titles and abstracts. Failure to meet the inclusion criteria led to the exclusion of studies. Study participants were patients with SCLC who had received PCI with HA.

Results

**Figure 1:**
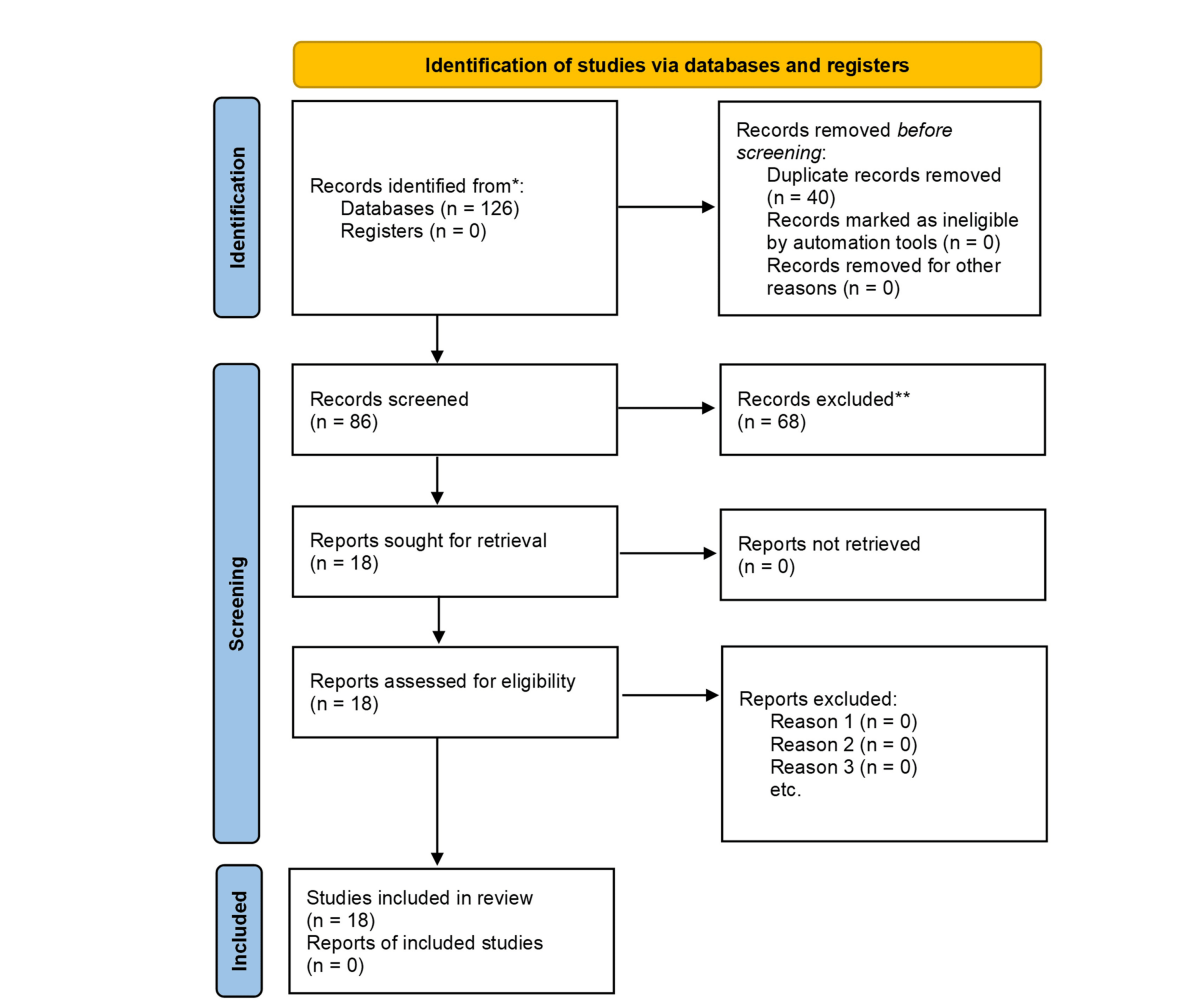
Preferred Reporting Items for Systematic Reviews and Meta-Analyses (PRISMA) flow diagram.

The search resulted in 126 articles, which were subsequently screened and selected based on their titles and abstracts. Of the 126 articles, 40 duplicate entries were removed, and 68 were excluded as they met our predefined exclusion criteria. Ultimately, 18 studies were included in the analysis, with their full texts retrieved and thoroughly assessed. In total, 18 studies published from 2007 to 2025 were included. Of these, two were phase III trials, two were prospective trials, one was a case report, and 13 were retrospective studies. The search and screening process outcomes are detailed in the PRISMA flow diagram presented in Figure 1 [16]. A comprehensive list of the articles selected for inclusion in this study is provided in Table 1.

**Table 1:** A comprehensive list of the articles included in the study and their outcomes. SCLC: small-cell lung cancer; WBRT: whole brain radiation therapy; PCI: prophylactic cranial irradiation; HA: hippocampal avoidance

Title	Authors	DOI	Year	Study type	Outcome
Prospective study of hippocampal-sparing prophylactic cranial irradiation in limited stage small cell lung cancer [17]	Kristin et al.	10.1016/j.ijrobp.2017.03.009	2017	Prospective study	This prospective study suggested a potential benefit in reducing neuropsychological sequelae of brain radiation, but at the cost of failures in the spared region
Risk of hippocampal metastases in small cell lung cancer patients at presentation and after cranial irradiation: a safety profile study for hippocampal sparing during prophylactic or therapeutic cranial irradiation [18]	Vijayananda et al.	10.1016/j.ijrobp.2014.12.026	2014	Retrospective study	The overall incidence of hippocampal metastasis before or after WBRT in SCLC patients is low. This provides preliminary support for the safety of hippocampal sparing during planned clinical trials of HA-WBRT for SCLC
Clinical features of brain metastases in small cell lung cancer: an implication for hippocampal sparing whole brain radiation therapy [19]	Guo et al.	10.1016/j.tranon.2016.11.00	2017	Retrospective study	Patients with multiple metastases are significantly associated with hippocampal and perihippocampal metastases. However, the incidence of perihippocampal disease may be acceptably low enough to perform hippocampal sparing-WBRT for SCLC
The risk of hippocampal metastasis and the associated high-risk factors in 411 patients with brain metastases [20]	Xie et al.	10.3389/fonc.2022.808443	2022	Retrospective study	Hippocampal sparing WBRT in SCLC patients with a greater number and total volume of metastases may result in a higher risk of tumor recurrence
Implications for preserving neural stem cells in whole brain radiotherapy and prophylactic cranial irradiation: a review of 2270 metastases in 488 patients [21]	Wan et al.	10.1093/jrr/rrs085	2012	Retrospective study	The incidence of involvement of the NSC regions is low, and the majority of NSC lesions were found in multi-metastatic patients. The study supported the selective reduction of doses for these structures in SCLC patients with oligometastatic disease with WBRT and patients with locally advanced stages of PCI
Distribution of brain metastases in relation to the hippocampus: implications for neurocognitive functional preservation [22]	Ghia et al.	10.1016/j.ijrobp.2007.02.016	2007	Retrospective study	A 5 mm safety margin around the hippocampus for conformal-sparing WBRT is an acceptable risk
Analyses of distribution and dosimetry of brain metastases in small cell lung cancer with relation to the neural stem cell regions: feasibility of sparing the hippocampus in prophylactic cranial irradiation [23]	Zhao et al.	10.1186/s13014-017-0855-3	2017	Retrospective study	A retrospective analysis that showed metastatic involvement of the neural stem cell regions (especially the hippocampus) is not common and is usually seen in patients with multiple mets in SCLC. Moreover, a dosimetric analysis showed that around 10% of patients may nevertheless have adequate dosage due to hippocampal PCI treatment
Perihippocampal failure after hippocampal avoidance whole-brain radiotherapy in cancer patients with brain metastases. Results of a retrospective analysis [24]	Shieh et al.	10.1097/MD.0000000000029144	2022	Retrospective study	A retrospective analysis on perihippocampal failure, which resulted two cases. The brain met could be attributed either to an under-dose or the aggressiveness of the tumor
Is hippocampal avoidance during whole-brain radiotherapy risky for patients with small-cell lung cancer? Hippocampal metastasis rate and associated risk factors [25]	Kirakli et al.	10.1177/1533034617742301	2017	Retrospective study	Hippocampal avoidance technique includes reducing the dose, which might be risky for hippocampal metastases in patients with SCLC in comparison to other malignant solid tumors
Estimation of intracranial failure risk following hippocampal-sparing whole brain radiotherapy [26]	Harth et al.	10.1016/j.radonc.2013.09.009	2013	Retrospective study	Prophylactic or therapeutic HS-WBRT is anticipated to carry a low risk of undertreatment. For SCLC, it presents a slightly increased risk of failure compared to standard WBRT. In NSCLC, HS-WBRT is unlikely to be linked with a clinically significant rise in the risk of failure
Distribution of metastasis in the brain in relation to the hippocampus: a retrospective single-center analysis of 565 metastases in 116 patients [27]	Sun et al.	10.1186/s40644-019-0188-6	2019	Retrospective study	This study demonstrated a low risk for perihippocampal metastases (PHM) and found no significant correlation between PHM and factors such as age, sex, KPS, primary site, total volume of intracranial metastases, or the entire brain. Therefore, excluding the perihippocampal region during WBRT may be reasonable
Intracranial failure after hippocampal‑avoidance prophylactic cranial irradiation in limited‑stage small‑cell lung cancer patients [28]	Cho et al.	10.1038/s41598-021-86851-6	2021	Retrospective study	For limited-stage SCLC, HA-PCI was not linked to either DFS or OS. While HA-PCI may be associated with a higher risk of intracranial failure, it did not negatively impact disease control or overall survival
Intracranial metastatic disease spares the limbic circuit: a review of 697 metastatic lesions in 107 patients [29]	Marsh et al.	10.1016/j.ijrobp.2009.02.038	2010	Retrospective study	It is appropriate to consider selectively excluding or lowering the dose to the limbic circuit when treating patients with prophylactic cranial irradiation or WBRT for oligometastatic disease that does not affect these areas
The incidence and location of brain metastases following HA-PCI compared with standard PCI in small cell lung cancer (SCLC): a phase III trial [30]	Belderbos et al.	10.1016/j.ijrobp.2019.06.450	2019	Phase III trial	This randomized phase III trial evaluated the safety of HA-PCI and compared the incidence and location of brain metastases following HA-PCI treatment to standard PCI. With a median follow-up of 24.6 months, there was no significant difference in the incidence of brain metastases between the standard PCI and HA-PCI groups. Additionally, no patients developed brain metastases in the hippocampus or the HA region
MA22.11 Risk of hippocampal metastases in small cell lung cancer: implications for hippocampal sparing cranial irradiation [31]	Effeney et al.	10.1016/j.jtho.2018.08.509	2018	Prospective study	The rate of hippocampal metastases is notably high in our SCLC patient cohort. Given that HS-WBRT may increase the risk of treatment failure in the spared area, prospective randomized trials are advised
Patterns of relapse following hippocampal avoidance prophylactic cranial irradiation for small cell lung carcinoma [32]	Cook et al	10.5603/RPOR.a2021.0119	2021	Retrospective study	In a series of 17 patients with SCLC with a complete (limited stage) or good partial (extensive stage), there were no hippocampal, only relapses. The study concluded that HA-PCI is a safe alternative to standard PCI in the setting of SCLC
Perihippocampal metastasis following hippocampus-avoiding prophylactic cranial irradiation for small cell lung cancer: a case report [33]	Yeo	10.2147/OTT.S143719	2017	Case report	A rare case that highlights the concerns about the potential for tumor recurrence near the hippocampus, even when efforts are made to spare this region during radiation therapy. Further research needs to confirm the clinical feasibility and safety of hippocampus-sparing approaches in treating SCLC patients
Randomized phase III trial of prophylactic cranial irradiation with or without hippocampal avoidance for small-cell lung cancer (PREMER): a GICOR-GOECP-SEOR study [34]	Rodriguez de dioz et al.	10.1200/JCO.21.00639	2021	Phase III trial	The study found that hippocampal avoidance during PCI significantly reduced the incidence of cognitive decline without compromising the treatment’s OS or efficacy in preventing brain metastases. These results suggest that HA-PCI could be a viable approach to protect neurocognitive functions in SCLC patients undergoing PCI

Discussion

According to scientific evidence, the benefit of PCI in SCLC patients is widely accepted. The incidence of brain metastases can be reduced, and the overall survival may also be improved. Given that neurocognitive function may be adversely affected by WBRT, many scientific efforts aim to reduce the potential radiation toxicity by implementing HA WBRT and PCI. However, by delivering this radiotherapy technique, there may be an increased risk of intracranial failure in the hippocampal region. Some data indicate a high risk of recurrence in the hippocampal region exceeding 10%, while other studies highlight that the risk remains low in this zone, estimated at <5%. In 2007, Ghia et al. tried to define the location of metastases related to the hippocampus by delivering different radiotherapy techniques (WBRT alone, stereotactic radiosurgery (SRS) alone, or WBRT and SRS) [22]. They analyzed data from 10 SCLC patients, where 24 brain metastases were detected. It was found that 12.5% of these metastases occurred within 5 mm of the hippocampus, and another 12.5% occurred between 10 mm and 15 mm from the hippocampus. Their results showed that the majority of brain metastases occur in an area more than 5 mm away from the hippocampus, concluding that a 5 mm margin around the hippocampus may be safe for HA PCI. Two years later, Marsh et al. published their review data about the incidence and location of brain metastases in 107 patients after retrospectively reviewing MRI scans [29]. Overall, 2.29% of all metastatic lesions occurred in the hippocampus, while specifically among SCLC patients, the hippocampus was involved in 2.1% of all lesions. As a result, the authors concluded that although the incidence of hippocampal involvement is low in oligometastatic patients (1-3 lesions), clinicians should pay attention to implementing hippocampal sparing in non-oligometastatic cases. Additionally, an interesting large review from Wan et al. concerned 488 patients with intracranial metastases after previous WBRT or PCI [21]. The hippocampal region failure involved only 0.8% of patients, while specifically regarding SCLC patients, 0.44% of lesions were found in the hippocampus. Given that 14.3% of hippocampal metastases were observed in patients with oligometastatic disease (1-4 lesions), while the vast majority occurred in non-oligometastatic patients, the authors concluded that radiotherapy techniques for both hippocampal and neural stem cell region sparing could be occasionally implemented in oligometastatic patients, due to the higher metastatic tendency in these regions among non-oligometastatic patients, extra caution is essential.

Moreover, Kundapur et al. (2014) reported a low incidence of recurrence in the hippocampal region in 20 SCLC patients who presented with brain metastases after WBRT [18]. Hippocampal metastases, which were characterized as lesions within 5 mm of the hippocampus, occurred in only one (5%) patient. In contrast, logistic regression analyses reported no correlated risk factors concerning hippocampal metastasis development. Due to the small sample size of their study, the authors declared that their findings must be evaluated with caution regarding the highly aggressive behavior of this malignancy. On the contrary, one year earlier, Harth et al. published the retrospective results of their study about the incidence of intracranial metastases after HA WBRT in 100 patients [26]. The study defined a 5 mm distance to the hippocampus as hippocampal metastasis. Among 11 SCLC patients, 18.2% of metastases occurred in the hippocampal region and 27.2% in the hippocampal +5 mm area, presenting a higher incidence rate than non-SCLC patients. However, in the entire sample, the incidence rate of hippocampal metastases was very low, estimated at 0.4%. Thus, in the case of HA WBRT, clinicians should pay attention to SCLC patients due to the higher tendency of recurrence in or near the hippocampus than in non-small-cell lung cancer patients. A few years later, Redmond et al. demonstrated the potential benefit of sparing the hippocampus in restricting neurocognitive impairment in 20 SCLC patients [17]. However, a relatively high risk of failure in the spared zone was documented. In the same year, Guo et al. retrospectively evaluated 180 SCLC patients, and 5 and 12.2% of patients presented hippocampal and perihippocampal metastases, respectively [19]. They declared that there is a higher risk of hippocampal metastases when the number of brain metastases is equal to or above five. At the same time, the incidence in the perihippocampal region was higher in the case of seven or more metastases.

Similarly, a case report from the scientific team of Yeo illustrated the necessity of further research given the HA PCI in SCLC patients [33]. One patient with SCLC underwent concurrent chemoradiotherapy and adjuvant HA PCI. After seven months of follow-up, a solitary brain metastasis occurred in the perihippocampal zone, defined as 5 mm inside the hippocampus, raising reasonable questions about the safety of HA PCI in these patients. The same year, Zhao et al. retrospectively evaluated the rate of brain metastases in 238 SCLC patients and found a low incidence (2%) within the subventricular zone. In contrast, only 1% were located in the HA region [23], as the perihippocampal zone was defined as the area within 5 mm of the hippocampus. Despite the rare metastatic involvement of neural stem cell regions, non-oligometastatic patients presented a higher rate within neural stem cell compartments (especially the hippocampus), highlighting the great concern about this category of patients. In addition, the scientific team of Kirakli et al. also reported their study outcomes about the intracranial failure of 54 patients after HA WBRT by retrospectively evaluating MRI scans [25]. In total, 17 (32%) patients had hippocampal metastases, and 4.4% were at the HA area, which was defined as 5 mm around the hippocampus. Among SCLC patients, 18.2% presented metastases in the hippocampal area, 27.3% within 5 mm, and 36.4% within 15 mm from the hippocampus. Consequently, according to their results, the authors illustrated that the HA WBRT in SCLC patients might raise a significant risk, given the high probability of perihippocampal failure.

In 2018, Effeney et al. presented interesting results in their cohort about the risk of hippocampal metastases after HA PCI in SCLC patients [31]. Indeed, they included 120 patients, of whom 44.2% had already presented brain metastases or developed them later without having previously received PCI. Among 53 patients who developed later brain metastases without previous PCI, hippocampal metastases occurred in 18.3%, while 3.1% involved the hippocampal area. The HA zone was defined according to the RTOG 0933 atlas as the area of 5 mm radial expansion from the hippocampus. This cohort reported a high incidence of intracranial failure in the hippocampal zone, highlighting the increased risk of HA PCI instead of standard PCI in SCLC patients. Their cohort concluded that SCLC patients present a high risk of failure in the HA region, and consequently, much caution is required. Moreover, data from a retrospective analysis of 116 patients published in 2019 showed a low incidence of metastasis in the hippocampal area [27]. Only 1.7% of lesions occurred in the hippocampus, while 11.2% were in a region within 5 mm around the hippocampus. Given that most lesions included the area outside the 15 mm surrounding the hippocampus, the study concluded that HA WBRT may be accepted and safely given.

Furthermore, in a recently randomized phase III clinical trial from Belderbos et al., 168 patients were randomized to receive PCI with or without HA [35]. Brain metastases were observed in 23 patients after a mean follow of 24.6 months; however, none were located on the hippocampal or perihippocampal region. However, the HA zone failed in one of 15 patients with multiple metastases. Finally, according to their abstract, no significant difference was observed between the two arms. Although larger studies with longer follow-ups are needed for the safety of HA PCI in SCLC patients, this study reported no statistically significant difference between common and HA PCI, given the failure in the perihippocampal zone.

In a further randomized trial by Cho et al., the same year, data from 126 patients who had received PCI with or without HA were retrospectively evaluated [28]. Out of 21 recurrent patients, 10 were in the HA PCI arm. No significant difference was observed, given the intracranial failure between the groups. However, after statistical analysis, patients with HA PCI showed a higher incidence of intracranial failure (HR = 2.87, 95% CI = 0.86-9.58, p = 0.087), and two of the patients in this group recurred in the perihippocampal region.

Moreover, another institution has recently published the results of their study regarding the incidence of intracranial failure in SCLC patients after PCI with or without HA [32]. After a mean follow-up of 11.6 months, 3 of 17 patients had multifocal relapses, including the HA zone, while no isolated relapse was observed in this zone. However, three non-oligometastatic patients had multiple relapses, including the perihippocampal zone. As a result, they represented the HA PCI as an equal alternative to the standard PCI method in SCLC patients. A randomized phase III trial also investigated the impact of using HA PCI compared to classical PCI in 150 SCLC patients on neurocognitive function and intracranial failure [34]. After a median follow-up of 40.4 months, an improved cognitive function was observed in the arm of hippocampal sparing; however, no significant difference was reported regarding the incidence of brain metastases, overall survival, and quality of life.

Furthermore, a recent retrospective study from 2022 by Xie et al. provided significant insights into the risk of failure in the hippocampal zone related to brain metastases. [20]. They retrospectively evaluated the data from 411 patients with brain metastases, measuring their distance from the hippocampus. The areas 5 mm, 10 mm, and 15 mm within the hippocampus had been defined. Given SCLC patients, they retrospectively reported a higher incidence rate of metastases in the hippocampal and perihippocampal zone (+5 mm) (18.1% and 22.9%, respectively) compared to other tumor types, highlighting the need for major concern about implementing HA PCI in SCLC patients.

Finally, a recent observational study examined the recurrence risk in the hippocampal zone after HA WBRT by retrospectively analyzing patients with brain metastases. Of the included 24 patients, 17 had lung cancer [24]. Although the perihippocampal failure rate was about 8%, only one case had the diagnosis of SCLC. Either the factor of underdose of radiation or the highly aggressive tumor behavior could justify the perihippocampal failure in some cases.

## Conclusions

SCLC constitutes one of the most aggressive types of neoplastic disease, a fact that raises major concerns regarding the most successful treatment strategy for this category of patients. Considering the improved outcomes of SCLC patients with PCI, much scientific interest is observed in the most successful intervention to achieve a dose reduction to the brain, thus its potential effects on cognitive functions. Consequently, the delivery of HA PCI is being rapidly implemented by an increased number of radiotherapy departments as an alternative option, with many questions about the potential risk of intracranial failure. Non-oligometastatic cases seem to be related to a higher incidence of hippocampal failure regions. In contrast, hippocampal-sparing techniques may be acceptable for SCLC patients, given the low incidence of metastases in the perihippocampal zone. Further data arising from the evaluation of HA PCI will enlighten the potential of this promising alternative strategy in reducing neurocognitive deficit problems. This could enhance the management of SCLC patients and improve their quality of life. Due to the lack of data, the design of more controlled clinical trials is required to assess this intervention’s beneficial role in the management of SCLC patients. Hence, new ideas based on this goal are considered fertile, leading to more appropriate therapeutic techniques for each patient.
